# Health, Secondhand Smoke Exposure, and Smoking Behavior Impacts of No-Smoking Policies in Public Housing, Colorado, 2014–2015

**DOI:** 10.5888/pcd13.160008

**Published:** 2016-10-20

**Authors:** Walter Young, Shelley Karp, Peter Bialick, Cindy Liverance, Ashley Seder, Erica Berg, Liberty Karp

**Affiliations:** Author Affiliations: Shelley Karp, Abacus Statistical Consultants, Portland, Oregon; Peter Bialick, GASP of Colorado, Boulder, Colorado; Cindy Liverance, Ashley Seder, American Lung Association in Colorado, Centennial, Colorado; Erica Berg, Denver Health and Hospital Authority, Denver, Colorado; Liberty Karp, research consultant, Portland, Oregon.

## Abstract

**Introduction:**

Exposure to secondhand smoke is problematic for residents living in multiunit housing, as the smoke migrates through shared ventilation systems, unsealed cracks, and door spaces. The objective of our research was to assess resident exposure to secondhand smoke, support for no-smoking policies, and the health impacts of no-smoking policies in multiunit housing.

**Methods:**

Surveys of 312 heads of households who resided in 1 of 3 multiunit buildings managed by a Colorado public housing authority were administered before and after implementation of a no-smoking policy that prohibited smoking in all resident apartments and all indoor common areas. A matched-pairs analysis of initial surveys and 15-month post-policy implementation surveys for 115 respondents was conducted.

**Results:**

Decreases were found in the number and percentage of smokers who smoked every day and the number of cigarettes smoked per day, and 30% had quit smoking 15 months after policy implementation. The percentage of residents who smelled secondhand smoke indoors declined significantly. A significant decrease in breathing problems was found after policy implementation. Although decreases were found in the incidence of asthma attacks, emphysema/chronic obstructive pulmonary disease, eye irritation, colds, nasal congestion, and ear/sinus infections, these decreases were not significant.

**Conclusion:**

Consistent findings across nearly all variables tested suggest that no-smoking policies reduce resident exposure to secondhand smoke, lower the incidence of secondhand smoke–associated breathing problems, decrease daily smoking and cigarette consumption, encourage smoking cessation, and increase quit attempts. If implemented in all multiunit housing, these policies could reduce exposure to secondhand smoke and health problems associated with secondhand smoke, promote smoking cessation, and reduce cigarette consumption.

## Introduction

A 2010 report of the US Surgeon General concluded that the following health consequences are among those that are causally linked to secondhand smoke exposure in adults: nasal irritation, coronary heart disease, middle ear disease, respiratory symptoms, impaired lung function, and lower respiratory illness (1). Exposure to secondhand smoke is particularly problematic for residents living in multiunit housing (MUH) who want to be smoke-free. Secondhand smoke migrates through shared ventilation systems, unsealed cracks, and door spaces. Half of MUH residents report that secondhand smoke enters their units from elsewhere (2), increasing nicotine concentrations in both smoking and nonsmoking homes (3).

Residents of public housing are typically low income (2), making it financially difficult for them to access smoking cessation help or move to avoid secondhand smoke. Although tobacco use has declined in the United States, smoking rates are still high among low-income and racial/ethnic minority populations (3). Because a greater percentage of low-income public housing residents are smokers (4), residents are more likely than the general population to have a neighbor who smokes and therefore be exposed to secondhand smoke. Most low-income residents in public housing say they prefer living in a nonsmoking building (4). Eighty-two percent of Colorado’s adults do not smoke, and 83% of Colorado households reported having smoke-free home rules (5). Protecting all residents by prohibiting smoking in public housing authority residential units is an opportunity to improve public health (6).

At least 8.7 million low-income residents live in US public housing (7). Health outcomes associated with secondhand smoke and the high cost of cleaning and turning over smoke-damaged residential units indicated a need for smoke-free policies in public housing over the past decade (8,9). In response to this growing evidence, the US Department of Housing and Urban Development (HUD) issued Housing Notice 2010–21, recommending that owners and management agents of multifamily housing adopt smoke-free housing policies. A recent HUD notice states, “Many owners and management agents (O/As) participating in one of the Multifamily Housing rental assistance programs listed in Section III of this Notice have taken steps to implement smoke-free housing policies in some or all of the properties they own/manage since the issuance of Housing Notice 2010-21” (6).

Using a national adult smoking prevalence estimate of 27.7% for households with annual incomes of less than $25,000 (1), we estimate that if smoke-free policies were adopted across all low-income public housing, 2.4 million adult smokers would be affected and thereby live in environments that are supportive of quitting or reducing their smoking habits. Another 6.3 million low-income nonsmokers would benefit from reduced exposure to secondhand smoke.

The objective of our research was to assess resident exposure to secondhand smoke, support for no-smoking policies, and the health impacts of no-smoking policies in MUH. Our pre-to-post policy impact research, approved by the Colorado Department of Public Health and Environment’s institutional review board, tested outcome variables associated with implementation of a no-smoking policy in an urban Colorado Public Housing Authority (PHA).

## Methods

The PHA implemented a no-smoking policy on December 1, 2013, in 3 buildings with 312 residential units. This policy contained a grandfather clause that permitted smoking for 12 months, only for resident smokers who requested and were granted a permit from PHA management. As of December 1, 2014, the policy had no exemptions and prohibited smoking in all indoor locations and on balconies and patios by residents, guests, and staff. PHA staff were responsible for monitoring compliance and enforcing the policy. Smoking was permitted only in designated outdoor locations that were at least 25 feet from doorways and windows.

In late February 2014, our baseline (T1) survey was mailed to all 312 heads of households to assess the 3-month period immediately before policy implementation. The follow up (T2) survey, administered in early March 2015 (approximately 1 year after T1), assessed a 3-month period beginning 12 months after the policy was implemented, after the grandfather period expired. The T1 and T2 surveys assessed changes in exposure to and sources of secondhand smoke; acute and chronic health problems associated with secondhand smoke (1); smoking behaviors — cessation, consumption, and frequency; and attitudinal measures before and after implementation of the no-smoking policy.

In cooperation with PHA managers, surveys were addressed generically to the “Head of Household” at each residential address. Gift cards were offered to compensate respondents for their time to complete the surveys. Completed surveys were logged in using a unique household code previously assigned to each survey form, to ensure anonymity and enable tracking of responses. Two follow-up mailings were sent to each nonresponding household to increase response rates. The data were cleaned and entered into a database for analysis.

Among data items included in the surveys were

- Month and year of birth, sex, race, and ethnicity, used for matching responses;
- Secondhand smoke exposure at home (source and location of exposure);
- Frequency and type of acute health events experienced in the last 3 months (allergies, chronic/acute lung disease, heart problems, headaches, eye irritation, nasal congestion, colds, and ear/sinus infections);
- Satisfaction with the no-smoking policy;
- Smoking status; and
- For smokers, current smoking behavior (eg, daily cigarette consumption, the number of quit attempts in past year).

We conducted matched-pairs analyses to assess head of household changes in the data items only from those heads of households who responded to both the T1 and T2 surveys, matched on residential unit number, month and year of birth, race, ethnicity, and sex to ensure that we had the same head of household at T1 and T2. By using a matched-pairs analysis we eliminated the possibility that respondents at T2 may have differed from respondents at T1, which would have confounded our findings.

Two statistical tests were used to assess changes from T1 to T2. In comparisons of multiple ordinal categories (eg, 5 categories of frequency of smelling secondhand smoke), the Wilcoxon matched-pairs signed rank test (10,11) was used. This nonparametric test was chosen to test differences that were not normally distributed. For data with dichotomous outcomes (eg, ever vs never smelling secondhand smoke or ever vs never experiencing a health issue), the McNemar test (12), a χ^2^ test for matched pairs, was used.

To identify smokers, we asked participants if they ever smoked 100 or more cigarettes in their lifetime and if they smoked at all in the previous 3 months.

## Results

Of 312 households, surveys from 156 (50%) households were completed at T1, and surveys from 168 (58%) households were completed at T2. Surveys from 115 (37%) households were completed at T1 and T2; only data from these were analyzed.

Approximately 76% of respondents were aged 60 or older, approximately 80% were female, and 90% were white, with each of the remaining race groups (black, Asian, and American Indian) representing approximately 2% of the respondents. Approximately 7% of respondents reported that they were Hispanic or Latino.

Eighteen respondents (16%) at T1 were smokers. All of them reported smoking every day. At T2, the number of daily smokers declined to 7 (39%); 5 (28%) smoked rarely to a few times per month, and 6 (33%) had quit smoking (half during the grandfather period and half after the policy was fully implemented). The change in frequency of smoking was significant (*P* = .01) (11,12).

The number of cigarettes smoked per day declined from T1 to T2, with 6 respondents not smoking any cigarettes at T2, 8 smoking less than half a pack at T1 and 7 at T2, and the number smoking at least half a pack a day decreasing from 9 to 5. The change in the number of cigarettes smoked each day was significant (*P* = .01).

At T2 we also asked smokers, “Compared to how much you smoked each day at the start of the no-smoking policy (December 1, 2013) how much do you smoke now?” Of the 18 smokers at T1, 6 had quit smoking. None of the 12 smokers at T2 reported smoking more cigarettes daily; 4 were smoking the same amount and 8 were smoking less than at T1. No one reported smoking more than a pack (>20 cigarettes) per day.

For the 12 smokers at T2, the number of reported quit attempts during the previous 12 months increased compared to the 12-month period before T1. Among these smokers, the number who made 2 or more attempts increased from 4 at T1 to 5 at T2, and the number who made no attempts decreased from 6 to 3.

A smaller percentage of heads of households reported smelling secondhand smoke at T2 than at T1 (Table 1), and they reported decreases in frequency of smelling secondhand smoke in their apartment from any source or in any indoor location where smoking was prohibited, including someone smoking in their own apartment (*P* < .001), from someone else’s apartment (*P* < .001), and from entryways and stairs or hallways (*P* < .001). A reduction in frequency of secondhand smoke exposure outdoors on porches, patios, or balconies was also reported (*P* = .04). The reduction in secondhand smoke exposure in apartments from outdoor sources was not significant.

**Table 1:** Location and Source of Smelling or Breathing Secondhand Smoke Reported by Study Respondents (n = 115) in the 3 Months Before (T1) and 13 to 15 Months After (T2) Implementation of a No-Smoking Policy in Public Housing Facilities, Colorado, 2014–2015

Location and Source	T1, n (%)	T2, n (%)	Wilcoxon *P* Value^a^	McNemar *P* Value^b^
Someone smoking in my apartment	36 (31.3)	14 (12.2)	<.001	.001
In my apartment from someone else’s apartment	67 (58.3)	45 (39.1)	<.001	.001
In my apartment from outside	60 (52.2)	56 (48.7)	.011	.12
In entryways, stairs, or hallways	90 (78.3)	73 (63.5)	*<*.001	*<*.001
Outdoors on porches, patios, or balconies	74 (64.3)	69 (60.0)	.04	.08
In the parking lot or on sidewalks	78 (68.8)	81 (70.4)	.39	.85

a Large-sample approximation to Wilcoxon test, comparing frequency, ranging from “every day” to “never” in the past 3 months.

b McNemar 2 × 2 test, comparing ever versus never smelling secondhand smoke in the past 3 months.

Support for the no-smoking policy was high overall (87% at T1 and 89% at T2) (Table 2). Twenty-six changed their responses, mostly in the direction of increased support, although these changes were not significant. For the nonsmokers, support was about 93% at T1 and T2. Of the 6 who quit smoking between T1 and T2, 4 were strong supporters of the policy at T2. For respondents who smoked, support was 55% at T1 and 50% at T2, with 17% strongly supporting it at T2.

**Table 2:** Support Among Study Respondents (n = 115) for the Building No-Smoking Policy 3 Months Before (T1) and 13 to 15 Months After (T2) Implementation of a No-Smoking Policy in Public Housing Facilities, Colorado, 2014–2015

Category/Period (n)	Strongly Support, n (%)	Somewhat/Slightly Support, n (%)	Do Not Support, n (%)	Don’t Know/Not Sure or No Response, n (%)
**All respondents**				
T1 (115)	81 (70.4)	19 (16.5)	9 (7.8)	6 (5.2)
T2 (115)	88 (76.5)	14 (12.2)	8 (7.0)	5 (4.3)
**Nonsmokers**				
T1 (97)	79 (81.4)	11 (11.3)	3 (3.1)	4 (4.1)
T2 (103)	86 (83.5)	10 (9.7)	3 (2.9)	4 (3.9)
**Smokers**				
T1 (18)	2 (11.1)	8 (44.4)	6 (33.3)	2 (11.1)
T2 (12)	2 (16.7)	4 (33.3)	5 (41.7)	1 (8.3)

About two-thirds of respondents indicated at both T1 and T2 that if they were moving to a new building it was very important to move to a nonsmoking building, and 85% said it was at least slightly important. Thirty-five respondents changed their responses from T1 to T2; among these, 60% said a nonsmoking building was more important, and 40% said it was less important. These differences were not significant.

Table 3 shows the frequency with which matched-pair respondents reported having health problems associated with exposure to secondhand smoke in the 3 months before each survey. Allergies and headaches each showed slight increases from T1 to T2, and heart problems remained constant; none of these changes was significant. The percentage of respondents reporting asthma attacks, emphysema/chronic obstructive pulmonary disease, eye irritation, colds, nasal congestion, and ear/sinus infections declined from T1 to T2. However, only breathing problems significantly improved in a comparison of ever versus never having these problems (11). No significant differences in frequency of having each health problem were detected for 5 categories (ranging from “every day” to “never”) from T1 to T2.

**Table 3:** Health Problems of Study Respondents (n = 115) During the 3-Month Period Before (T1) and 13 to 15 Months After (T2) Implementation of a No-Smoking Policy in Public Housing Facilities, Colorado, 2014–2015^a^

Secondhand Smoke–Associated Health Problems	T1, n (%)	T2, n (%)	Wilcoxon *P* Value^b^	McNemar *P* Value^c^
Asthma attacks	22 (19.1)	17 (14.8)	.42	.17
Emphysema/chronic obstructive pulmonary disease	23 (20.0)	20 (17.4)	.48	.37
Heart problems	23 (20.0)	23 (20.0)	.58	>.99
Allergies	53 (46.1)	56 (48.7)	.19	.58
Breathing problems	58 (50.4)	47 (40.9)	.65	.03
Headaches	60 (52.2)	65 (56.5)	.54	.42
Eye irritation	69 (60.0)	66 (57.4)	.26	.63
Nasal congestion	78 (67.8)	69 (60.0)	.15	.12
Colds	63 (54.8)	61 (53.0)	.77	.77
Ear/sinus infections	51 (44.4)	42 (36.5)	.47	.15

a Respondents were asked about the frequency of health problems within the 3 months before T1 and T2.

b Large-sample approximation to Wilcoxon test, comparing frequency, ranging from “every day” to “never” in the past 3 months.

c McNemar 2 × 2 test, comparing ever versus never smelling secondhand smoke in the past 3 months.

## Discussion

We found that the no-smoking policy was associated with people quitting smoking, reductions in every day smoking and the number of cigarettes smoked per day, and an increase in 2 or more quit attempts in the 12 months before T2 compared to 12 months before T1. Also, no one reported smoking more than a pack per day in the previous 3 months at T2. These findings suggest that for residents of public housing who smoke, no-smoking policies are an important factor in motivating quit attempts, reducing cigarette consumption, and promoting smoking cessation. These findings are consistent with recommendations from a systematic search of peer-reviewed articles that concluded that “future research on smoke-free MUH policy . . . on the impact of these policies on smoking behaviours and health outcomes could further inform public health planning, policy and practice” (13).

We found that the percentage of heads of households who ever smelled secondhand smoke indoors, as well as the frequency of smelling it, decreased after full implementation of the no-smoking policy for every building location except for smelling smoke in apartments from outside. Frequency of secondhand smoke exposure outdoors from smoking on balconies, patios, and porches also decreased. No-smoking policies in public housing can be an important tool for reducing resident exposure to secondhand smoke. Other studies arrived at the same conclusion (14–16).

Perhaps our most important public health finding was the reduction in the incidence of acute breathing problems associated with smelling or exposure to secondhand smoke. Our study also found that all of the other health conditions associated with exposure to secondhand smoke tended to improve, although not significantly. Our literature search did not find studies that asked about the health outcomes associated with smoking bans in public housing. Future studies are recommended to test the relationship between no-smoking policies in low-income housing and public housing and health outcomes.

An important indicator of the success of this policy was the reported support for the no-smoking policy among smokers and nonsmokers alike. The high level of support suggests that adoption of these policies would be welcomed among MUH residents. Our findings confirmed the support reported in most other studies, but some of those studies showed less support among smokers than ours. One study found that overall, 74% of low-income tenants in a group of subsidized, multiunit buildings were “very” or “somewhat” happy with the smoke-free policy, but only 30% of current smokers were happy with the policy (17), whereas we found that better than 90% overall, and half of the smokers, supported the policy. Another study found that most nonsmokers (79%) preferred that their buildings be smoke-free (4).

The study results are limited by a few factors. First, the study was conducted on residents of a single public housing organization, representing 3 multiunit buildings of households occupied primarily by seniors. Second, the small number of smokers made it difficult to statistically test outcomes. Ideally, the baseline survey would have been administered immediately before implementation of the policy and the follow-up would have been after the policy had been in effect for a longer period of time, preferably with no grandfather period allowing residents more time to quit smoking.

The reductions in reported exposure to indoor secondhand smoke from before policy implementation to after, the increase in 2 or more quit attempts annually, the declines in the frequency of smoking and number of cigarettes smoked per day, the finding that many smokers quit smoking during policy implementation, and the decrease in secondhand smoke–associated health problems all suggest that these policies are highly effective in reducing smoking, exposure to secondhand smoke, and secondhand smoke–associated health problems. These findings are supported by a study that found that a smoke-free policy in workplaces not only protected nonsmokers from the dangers of passive smoking but also encouraged smokers to quit or to reduce consumption (18).

## References

[1] How tobacco smoke causes disease: the biology and behavioral basis for smoking-attributable disease: a report of the Surgeon General. Atlanta (GA): Centers for Disease Control and Prevention, Office on Smoking and Health; 2010. http://www.ncbi.nlm.nih.gov/books/NBK53017. Assessed December 22, 2015. 21452462

[2] Hewett MJ , Sandell SD , Anderson J , Niebuhr M . Secondhand smoke in apartment buildings: renter and owner or manager perspectives. Nicotine Tob Res 2007;9(Suppl 1):S39–47. 10.1080/14622200601083442 17365725

[3] King BA , Travers MJ , Cummings KM , Mahoney MC , Hyland AJ . Prevalence and predictors of smoke-free policy implementation and support among owners and managers of multiunit housing. Nicotine Tob Res 2010;12(2):159–63. 10.1093/ntr/ntp175 19959570 PMC3436436

[4] Hennrikus D , Pentel PR , Sandell SD . Preferences and practices among renters regarding smoking restrictions in apartment buildings. Tob Control 2003;12(2):189–94. 10.1136/tc.12.2.189 12773730 PMC1747712

[5] Van Deusen A , Hyland A , Travers MJ , Wang C , Higbee C , King BA , Secondhand smoke and particulate matter exposure in the home. Nicotine Tob Res 2009;11(6):635–41. 10.1093/ntr/ntp018 19351784

[6] US Department of Housing and Urban Development. Notice H 2012-22: further encouragement for O/As to adopt optional smoke-free housing policies. 2012. http://portal.hud.gov/hudportal/documents/huddoc?id=12-22hsgn.pdf. Accessed December 15, 2015.

[7] Lee D , Turner N , Burns J , Lee T . Tobacco use and low-income African Americans: policy implications. Addict Behav 2007;32(2):332–41. 10.1016/j.addbeh.2006.05.002 16828978

[8] Poppe H . Smoke-free apartments: it is time for a new amenity in multi-unit housing? CAA Perspective Magazine 2005;(May–July):30–2.

[9] Minnesota Smoke-free Housing. Damage from cigarette smoke residue: the cost of allowing smoking in multi-housing, January 2014. http://www.mnsmokefreehousing.org/fckfiles/Contemplation%20-%20Smoking%20Residue%20Damage(2).pdf. Accessed September 22, 2017.

[10] Daniel WW . Applied nonparametric statistics. Boston (MA): Houghton Mifflin Company; 1978; p. 31–5.

[11] Social Science Statistics. Wilcoxon signed-rank test calculator. http://www.socscistatistics.com/tests/signedranks/Default2.aspx. Accessed June 7, 2016.

[12] Fleiss JL . Statistical methods for rates and proportions. Second edition. New York (NY): John Wiley and Sons; 1981; p. 112–9.

[13] Snyder K , Hassett Vick J , King BA . Smoke-free multiunit housing: a review of the scientific literature. Tob Control 2015;25(1):9–20. 10.1136/tobaccocontrol-2014-051849 25566811 PMC4778954

[14] King BA , Travers MJ , Cummings KM , Mahoney MC , Hyland AJ . Secondhand smoke transfer in multiunit housing. Nicotine Tob Res 2010;12(11):1133–41. 10.1093/ntr/ntq162 20889473 PMC3436457

[15] Wilson KM , Klein JD , Blumkin AK , Gottlieb M , Winickoff JP . Tobacco-smoke exposure in children who live in multiunit housing. Pediatrics 2011;127(1):85–92. 10.1542/peds.2010-2046 21149434

[16] Brownson RC , Eriksen MP , Davis RM , Warner KE . Environmental tobacco smoke: health effects and policies to reduce exposure. Annu Rev Public Health 1997;18(1):163–85. 10.1146/annurev.publhealth.18.1.163 9143716

[17] Drach LL , Pizacani BA , Rohde KL , Schubert S . The acceptability of comprehensive smoke-free policies to low-income tenants in subsidized housing. Prev Chronic Dis 2010;7(3):A66. http://www.cdc.gov/pcd/issues/2010/may/09_0209.htm. Accessed May 5, 2016. 20394705 PMC2879998

[18] Fichtenberg CM , Glantz SA . Effect of smoke-free workplaces on smoking behaviour: systematic review. BMJ 2002;325(7357):188. 10.1136/bmj.325.7357.188 12142305 PMC117445

